# Adverse childhood experiences growing up in East or West Germany or abroad

**DOI:** 10.3389/fpsyt.2022.908668

**Published:** 2022-09-21

**Authors:** Ann-Christin Schulz, Christoph Kasinger, Manfred Beutel, Jörg M. Fegert, Vera Clemens, Elmar Brähler

**Affiliations:** ^1^Department of Psychosomatic Medicine and Psychotherapy, University Medical Center, Johannes Gutenberg University of Mainz, Mainz, Germany; ^2^Department of Psychiatry and Psychotherapy, University of Leipzig Medical Center, Leipzig, Germany; ^3^Department of Child and Adolescent Psychiatry and Psychotherapy, Medical Faculty of the University of Ulm, Ulm, Germany

**Keywords:** adverse childhood experiences, immigration, socio-political context, child abuse, child neglect, East and West Germany

## Abstract

**Background:**

Adverse childhood experiences (ACEs) are potentially traumatic events that occur before the age of 18. The term encompasses various adverse childhood experiences, e.g., physical, psychological, and sexual abuse, physical and psychological neglect, and family dysfunction. Prevalence estimates for a broad spectrum of ACEs against the background of where childhood and adolescence were spent are scarcely available in Germany. This study examines the frequencies of adverse childhood experiences, considering growing up in East or West Germany or abroad and interacting with different age cohorts and gender.

**Methods:**

A total of 5,018 individuals (51.4% female) aged 14 years and older were retrospectively assessed on adverse childhood experiences using questionnaires “adverse childhood experiences” (ACE). Logistic regression models were used to analyze the association between birth cohort, gender, and where a person grew up. Descriptive statistics and univariate analyses were used to calculate frequencies, proportions, and unadjusted associations for each variable.

**Results:**

37.4% (*N* = 1,878) of respondents reported experiencing at least one form of ACE. Individuals who grew up abroad report significantly more adverse childhood experiences than individuals in East or West Germany. Men and women who grew up in East Germany reported a lower rate of ACEs. We found significant effects for all predictors: Where childhood and adolescence were predominantly spent, year of birth, and gender. Significant differences in the prevalence of adverse childhood experiences within the gender groups were only found for sexual and physical abuse and substance dependence in the household.

**Conclusion:**

The results suggest that the socio-political context plays an essential role in the experience of adverse childhood experiences, both in frequency and risk. Thus, child abuse and neglect studies should increasingly focus on societal risk and protection mechanisms.

## Introduction

Adverse childhood experiences (ACE) are potentially traumatic events that occur in the age of 0–17 years and include abuse and neglect and household dysfunction (1). The manifestations of ACEs are manifold and include physical, sexual, or emotional abuse, physical and psychological neglect, and family dysfunction such as domestic violence or separation from a parent. ACEs can negatively impact health and life satisfaction, cause substance use in adulthood, and reduce career opportunities and earning potential (1, 2).

The consequences and effects are diverse: Somatic symptoms, general health status, and mental illnesses are among the consequences of ACEs, as well as social consequences for affected individuals (3). These include chronic pain, musculoskeletal disorders, obesity, diabetes, cancer, and stroke (4–6). ACEs have been associated with mental illnesses such as depression, anxiety, sleep and eating disorders, substance-related disorders, personality disorders, and post-traumatic stress disorder (7). In addition, ACEs are associated with a tenfold increased risk for suicide attempts, alcohol, and drug abuse (8–10). Importantly, persons who have experienced maltreatment during childhood are at higher risk of maltreating their children (11). According to Fox et al. (12), each additional negative experience leads to a >35% likelihood of becoming violent and delinquent in adolescence. International meta-analyses show high prevalence rates of 22.6% (85% CI: 20.3–25.1) for physical abuse, 36.3% (85% CI: 30.2–42.9) for emotional abuse, and 7.6% (85% CI: 6.4–8.5) for sexual abuse of boys and 18.0% (85% CI: 16.9–19.2) of girls worldwide (13). In a national study, 4.3% (95% CI: 3.5–5.1) of respondents reported experiencing childhood sexual abuse. Parental divorce/separation 19.4% (95% CI: 17.7–20.9) and alcohol and drug abuse in the home 16.7% (95% CI: 15.1–18.3) were among the most commonly reported household dysfunction (14). However, it should be noted that prevalence varies internationally (13); this indicates the political-social context.

Quite a few studies deal with a victim-perpetrator reversal and the assumption of transgenerational transmission of trauma, i.e., the transmission of experiences of the members of one generation to the members of a subsequent generation (11). However societal, and legal-preventive structures play an equally crucial role, especially in relation to neglect and emotional abuse. For example, a German study on authoritarianism and the transgenerational transmission of corporal punishment shows that participants who affirmed authoritarian submission were significantly more likely to endorse a slap or spanking (15).

For instance, recent studies show that social policies can increase and decrease child maltreatment rates (16, 17). For example, economic guidelines can reduce household income, which may be associated with increased maltreatment rates (18, 19). Results show that discrimination against women, gender inequality, and disparities can be associated with adult advocacy to hit a child with an object (severe physical abuse) (17). Conversely, women's positively impacts child development (17, 20, 21). A study by Fiala and LaFree (22) shows that countries that offer women more opportunities, such as tertiary education, have lower rates of child abuse. Gartner (23) also shows that disempowered women can less protect their children from third-party abuse. Policies such as paid parental leave, earned income tax credits, increases in the minimum wage, and more generous welfare benefits have also reduced child maltreatment (24, 25). Limited options thus create more stress and frustration for women, which can impact violence in childrearing.

With its divided past of two different countries but shared history and culture, Germany is an excellent research subject to investigate the consequences of different socio-political environments. The divided past begins with the end of the Second World War in 1945 and the Potsdam Agreement, which was decided in August 1945 to divide Germany into four occupation zones. The three western zones became democratic, and free elections took place. In the Soviet-administered zone, a Soviet-dependent communist dictatorship developed: the GDR (German Democratic Republic). Although per capita income was higher in West Germany than in neighboring East Germany, the economic and societal inequality was higher in West Germany (26). Despite the constitution's stipulation of gender equality, the GDR, in particular, failed to implement it (27). In East Germany, for example, both genders had the same right to work, which was made possible by the government through out-of-home childcare from an early age (28). State differences in the legal framework of child-rearing should also be mentioned; While corporal punishment of children was still considered acceptable in West Germany until 1973, it was banned in East Germany as early as 1949 with the founding of the state (29).

Reunification in 1989 was followed by a period of individualization and autonomy. The remaining social inequalities were relativized, and life forms and lifestyles seemed freely selectable (30). Despite the attempt to equalize living conditions after reunification, there is still a considerable difference between the levels of living in East and West Germany (31).

Studies already show the socio-political context as a determinant of child abuse (32, 33). However, our study also included individuals who did not spend their childhood and adolescence in Germany and was therefore considered a comparison group independent of German history.

The present study aims to shed light on underlying socio-political mechanisms for ACEs by investigating the prevalence of ACEs depending on where most of the childhood (East or West Germany or abroad) was spent. In doing so, this work focuses on the undercurrents of different types of abuse and neglect and other adverse childhood experiences. Furthermore, we are also considering the interaction of different age cohorts and gender.

## Materials and methods

The independent Institute for Opinion and Social Research (USUMA, Berlin) conducted the nationwide data collection. A total of two representative samples were examined. The first data collection occurred between February and March 2020, followed by April and June 2020. In addition to sufficient German language skills, a minimum age of 14 years was required for participation. The sample, which was representative of age, gender, education, and region of residence, was selected using the random route method with a default starting address. Using a specific street and house number, every third household was personally contacted by study staff and invited to participate in the study through interviews. In households with more than one person, the selection was made through the Kish selection grid, a random selection of respondents in multi-person households.

Information about the study and consent to participate was obtained from all participants by the interviewers. The study staff first collected sociodemographic information in a personal interview.

Subsequently, the participants were given a questionnaire to be completed independently and returned to the interviewers in a sealed envelope. The interviewers were not in the room but could help with comprehension problems. In the subsequent questionnaire analysis, the previously collected demographic data was linked to the completed questionnaire without names or other identifying information. The study was conducted by the Declaration of Helsinki and approved by the Ethics Committee of the Medical Faculty of Leipzig University.

### Sample description

A total of 5,018 people from two representative surveys (*N* = 2,503; *N* = 2,515) were included in the study. 4,935 were over 18 years of age, *N*). Participants were on average 49.93 years old [standard deviation (SD) = 17.78] of whom 2,578 (51.4%) were women. A large proportion of respondents reported having grown up predominantly in West Germany 3,711 (74.0%). Study participants who grew up predominantly abroad were underrepresented in this sample, at 5.5%, compared with the general population (proportion of the population with a migration background as of 1.10.2021 26.7%). Detailed information on the sample is presented in Table 1.

**Table 1:** Sample description.

	**Average (SD)**
**Age (years)**
Male	49.60 (17.58)
Female	50.25 (17.97)
**Quantity (valid %)**	
**Gender**
Male	2,440 (48.6)
Female	2,578 (51.4)
**Living with a partner**
Yes	2,182 (43.5)
No	2,823 (56.2)
**Nationality**
German	4,831 (96.3)
Not German	184 (3.7)
**Highest level of education**
No school-leaving qualification	109 (2.2)
School-leaving qualification	3,440 (68.5)
(Technical) Baccalaureate	830 (16.5)
University/technical college degree	531 (10.6)
**Monthly household income**
< 1,250 €	635 (12.6)
1,250–2,500 €	2,030 (40.5)
>2,500 €	2,260 (45.0)
**Predominantly grown up**
West Germany	3,711 (74.0)
East Germany	1,015 (20.2)
Abroad	276 (5.5)
**Age cohorts**
Until 1945	469 (9.3)
1946–1967	1,912 (38.1)
1968–1989	1,778 (35.4)
From 1990	859 (17.1)

### Instruments

Sociodemographic data such as age, gender, marital status, employment, and school-leaving qualifications were collected from all participants. In order to record where childhood and adolescence were spent, participants indicated in which country they predominantly grew up or in which federal state (The GDR comprised the territory of the present-day states of Brandenburg, Mecklenburg-Western Pomerania, Saxony, Saxony-Anhalt, and Thuringia).

Various forms of adverse childhood experiences were assessed using the German version of the Adverse Childhood Experiences Questionnaire. The questionnaire comprised 10 items and was first used by Felitti et al. (34). Each item represents one form of ACE: emotional, physical, and sexual abuse, emotional and physical neglect, separation from a parent, violence against the mother, substance dependence in the household, mental illness, and incarceration of a household member. The questions were answered “yes” and “no” on a dichotomous response scale and were for sexual abuse, for example: Did an adult or person at least 5 years older ever touch or fondle you in a sexual way or cause you to touch their body in a sexual way? Or attempted to have or had oral, anal, or vaginal intercourse with you?”

### Statistical analyses

Statistical analyses were performed using R, version 4.1.0 (35) and the dplyr (36), haven (37), and ggplot2 (38) packages and SPSS version 27. Descriptive calculations were used to determine the prevalence rates of each ACE. The Chi-square test was used to compare categorical variables, including demographic and ACE data between groups. Group comparisons were made by gender and ethnicity. We also examined prevalence rates in different age cohorts to account for social phenomena.

A binary logistic regression model was applied to determine independent relationships between gender, origin (West-East Germany or abroad), and age. The reference categories were male sex, predominantly those who grew up in West Germany, and the age cohort born before 1945. A *p*-value of < 0.05 was considered statistically significant.

## Results

### Frequencies of adverse childhood experiences

Overall, *N* = 1,815 (37.3%) reported at least one ACE, and 493 (5.5%) participants reported experiencing four or more ACEs.

Specifically, 1,533 (30.5%) participants reported experiencing emotional and/or physical and/or sexual abuse, and *N* = 903 (18.0%) reported experiencing emotional and/or physical neglect. Loss of a parent through death or separation was experienced by *N* = 949 (18.9%). Family dysfunction such as domestic violence, substance abuse, or mentally ill persons in the household, as well as whether a family member had been incarcerated, were affirmed by 1,604 (31.9%) participants.

Table 2 provides an overview of the cohorts measured by the number of ACEs experienced. For example, 42 (15.5%) of the predominantly foreign-raised respondents reported having experienced more than four ACEs, significantly more than the comparison group. The trend that foreign-born reported more ACEs runs through all age cohorts, except for the 1968–1989 age cohorts and those born after 1990 who grew up predominantly in East Germany: For example, over half (56.1%) of those who grew up in East Germany and were born in 1968–1989 reported having experienced at least one ACE. In comparison, those who grew abroad report less than half, 43.5%. In the cohort from 1990 onwards, the number of respondents who had at least four adverse childhood experiences was highest, with *N* = 15 (10.8%) of respondents who grew up predominantly in eastern Germany.

**Table 2:** Frequencies of adverse childhood experiences in West-East Germany in persons predominantly raised abroad (number *N* and corresponding percentage).

	**Number ACE**	**Total**	**West Germany**	**East Germany**	**Abroad**
Total		*N* = 4,892	*N* = 3,637	*N* = 984	*N* = 271
	0	3,077 (62.7)	2,193 (60.3)	736 (74.8)	142 (52.4)
	1	741 (15.2)	568 (15.6)	122 (12.4)	42 (15.5)
	2	327 (6.7)	269 (7.4)	34 (3.5)	23 (8.5)
	3	270 (5.5)	220 (6.0)	28 (2.8)	22 (8.1)
	>4	493 (10.1)	387 (10.6)	64 (6.5)	42 (15.5)
Cohort until 1945		*N* = 456	*N* = 302	*N* = 136	*N* = 17
	0	285 (62.5)	184 (60.9)	91 (66.9)	10 (58.2)
	1	80 (17.5)	44 (14.6)	31 (22.8)	4 (23.5)
	2	23 (5.0)	18 (6.0)	5 (3.7)	0 (0.0)
	3	20 (4.4)	18 (6.0)	1 (0.7)	1 (5.9)
	>4	48 (10.5)	38 (12.6)	8 (5.9)	2 (11.8)
Cohort 1946–1967		*N* = 1,862	*N* = 1,349	*N* = 413	*N* = 93
	0	1,178 (63.2)	821 (60.9)	311 (75.3)	44 (47.3)
	1	273 (14.6)	207 (15.3)	48 (11.6)	13 (14.0)
	2	123 (6.6)	99 (7.3)	14 (3.3)	10 (10.7)
	3	100 (5.4)	76 (5.6)	15 (3.6)	9 (6.7)
	>4	188 (10.1)	146 (10.8)	25 (6.0)	17 (18.3)
Cohort 1968–1989		*N* = 1,748	*N* = 1,310	*N* = 296	*N* = 138
	0	1,088 (62.2)	777 (59.,3)	130 (43.9)	78 (56.5)
	1	240 (13.7)	191 (14.6)	30 (10.1)	18 (13.0)
	2	120 (6.9)	98 (7.5)	11 (3.7)	11 (8.0)
	3	105 (6.0)	86 (6.6)	9 (3.0)	10 (7.2)
	>4	195 (11.2)	158 (12.1)	16 (5.4)	21 (15.2)
Cohort from 1990		*N* = 842	*N* = 676	*N* = 139	*N* = 23
	0	526 (62.5)	411 (60.8)	104 (74.8)	10 (43.5)
	1	148 (17.6)	126 (18.6)	13 (9.3)	7 (30.4)
	2	61 (7.2)	54 (8.0)	4 (2.9)	2 (8.7)
	3	45 (5.3)	40 (5.9)	3 (2.2)	2 (8.7)
	>4	62 (7.4)	45 (6.7)	15 (10.8)	2 (8.7)

### Frequencies of adverse childhood experiences in West and East Germany and abroad in gender comparison

**Figure 1:**
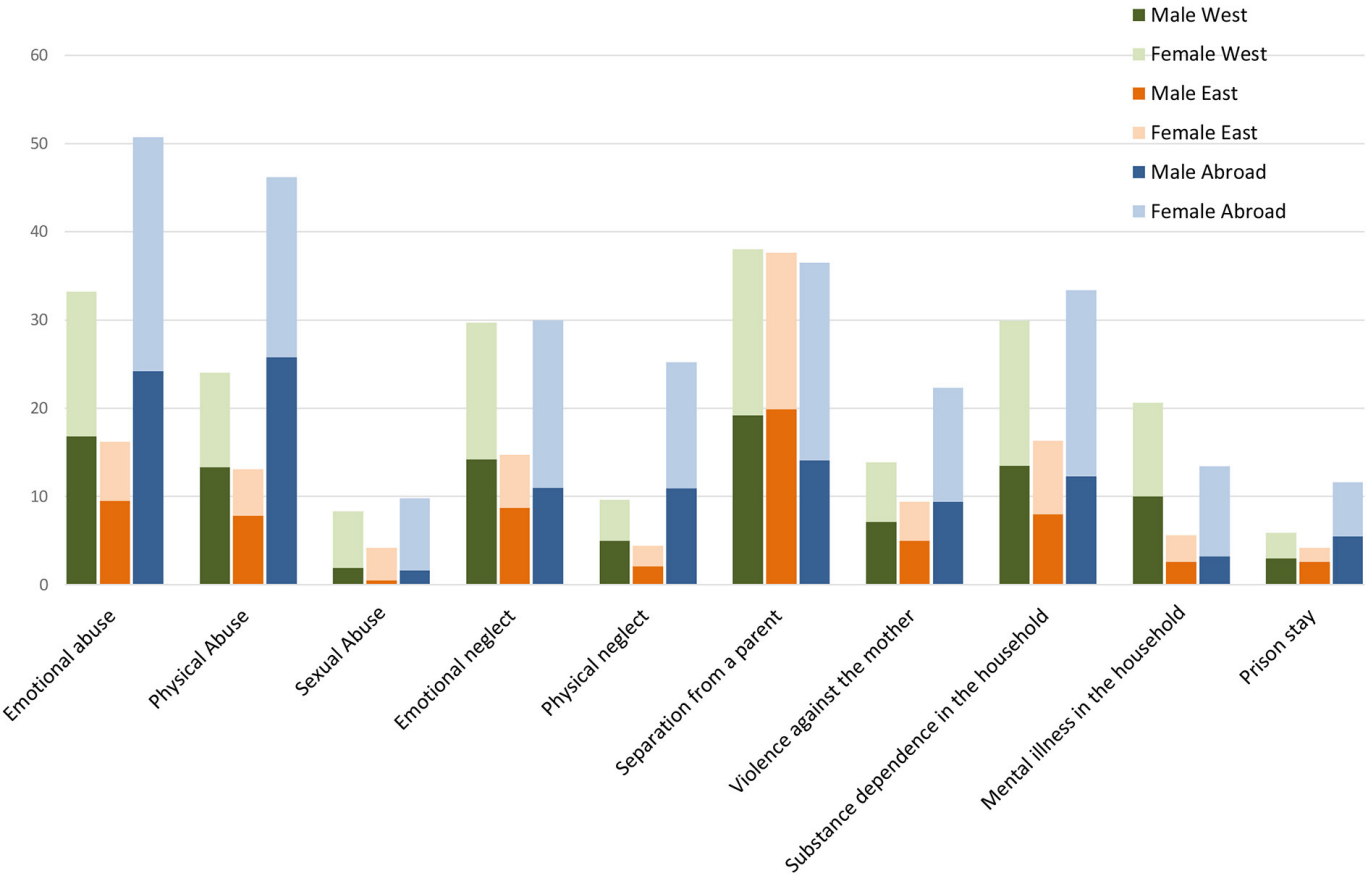
Relative prevalence (percentage).

The frequencies of the various ACEs in a gender comparison are shown in Table 3. Figure 1 illustrates the differences between West and East Germany and other countries.

**Table 3:** Relative frequency (percent) of individual adverse childhood experiences in West and East Germany and abroad and by gender (male, female).

	**Male**				**Female**				**Total**				**Total**		
	**West**	**East**	**Abroad**	**χ^2^**	**West**	**East**	**Abroad**	**χ^2^**	**West**	**East**	**Abroad**	**χ^2^**	**Male**	**Female**	**χ^2^**
Emotional abuse	16.8	9.5	24.2	25.47***	16.4	6.7	26.5	41.266***	16.6	8.3	25.5	64.70***	15.4	15.3	0.02
Physical abuse	13.3	7.8	25.8	32.81***	10.7	5.3	20.4	28.42***	11.9	6.7	22.9	58.66***	12.6	10.3	6.56
Sexual abuse	1.9	0.5	1.6	5.12	6.4	3.7	8.2	5.95*	4.3	1.9	5.1	13.72**	1.5	6.0	68.80***
Emotional neglect	14.2	8.7	11.0	12.20**	15.5	6.0	19.0	29.39***	14.9	7.5	15.3	37.65***	12.7	14.1	2.04
Physical neglect	5.0	2.1	10.9	20.47***	4.6	2.3	14.3	35.27***	4.8	2.2	12.7	53.89***	4.6	4.8	0.05
Separation from a parent	19.2	19.9	14.1	2.32	18.8	17.7	22.4	1.60	19.0	18.9	18.5	0.03	19.2	18.8	0.10
Violence against the mother	7.1	5.0	9.4	4.45	6.8	4.4	12.9	12.74**	7.0	4.8	11.3	15.51***	6.7	6.7	0.00
Substance dependence in the household	13.5	8.0	12.3	13.54**	16.4	8.3	21.1	22.23***	15.0	8.1	18.5	37.55***	12.3	15.3	9.45**
Mental illness in the household	10.0	2.6	3.2	36.59***	10.6	3.0	10.2	24.06***	10.3	2.8	7.0	57.89***	7.9	9.3	2.95
Prison stay	3.0	2.6	5.5	2.97	2.9	1.6	6.1	8.11*	2.9	2.2	5.8	10.07**	3.0	2.8	0.11

***p < 0.001, **p < 0.01, *p < 0.05.

Women reported more often adverse childhood experiences than men. Statistically significant differences between men and women were only found in the experience of sexual abuse χ^2^(1) = 68.80, *p* =< 0.001, and in the question of whether someone from the household had alcohol problems or used drugs, χ^2^(1) = 9.45, *p* =< 0.002.

Separation of parents is the most frequently reported ACE in Germany and the only item that does not register a significant difference between West and East Germany. Differences in gender perception are also evident in the experience of violence against the mother. Male participants report fewer experiences than women, and there are no differences between participants who grew up in West or East Germany or those who spent most of their childhood and youth abroad. The frequency of experiences of violence against the mother is higher among women who grew up abroad than among the female comparison groups χ ^2^(2) = 12.74, *p* =< 0.002.

### Frequencies of adverse childhood experiences in West and East Germany and abroad in the different age cohorts

Table 4 shows differences between the various age cohorts. The age cohort born up to 1945 represents the war generation of the Second World War, which ended on September 2^nd^, 1945. The second cohort, 1946–1967, is considered the post-war generation. Also significant is the founding of the GDR on October 7^th^, 1949. The third cohort, 1968–1989, begins with times of upheaval and worldwide protests and ends with the fall of the Berlin Wall on October 3rd, 1989. The third cohort, born after 1990, was raised in the period of numerous political changes and German reunification.

**Table 4:** Relative frequency (percent) of individual adverse childhood experiences in West and East Germany and abroad and age cohorts.

	**Until 1945**				**1946–1967**				**1968–1989**				**From 1990**			
	**West**	**East**	**Abroad**	**χ^2^**	**West**	**East**	**Abroad**	**χ^2^**	**West**	**East**	**Abroad**	**χ^2^**	**West**	**East**	**Abroad**	**χ^2^**
Emotional abuse	19.4	7.0	11.1	11.86**	18.4	8.7	34.0	42.30***	16.6	6.3	23.0	26.96***	11.8	12.8	16.7	0.59
Physical abuse	16.8	6.3	11.1	9.23**	13.2	7.7	28.7	31.51***	11.3	4.3	20.1	26.04***	8.3	9.2	25.0	0.00*
Sexual abuse	2.6	0.7	–	2.27	4.5	2.1	5.3	5.23	4.8	2.0	4.3	4.76	3.6	2.1	12.5	6.30*
Emotional neglect	15.5	7.9	11.1	4.96	15.0	8.0	17.0	14.68***	16.3	6.7	13.0	18.77***	11.7	7.8	25.0	6.25*
Physical neglect	9.2	6.4	16.7	2.50	4.9	2.3	13.8	22.45***	4.4	1.0	11.5	24.88***	3.2	–	12.5	12.14**
Separation from a parent	13.2	29.4	16.7	17.14***	16.0	16.3	21.3	1.79	22.1	17.6	18.7	3.51	21.4	19.3	8.3	2.61
Violence against the mother	9.5	3.5	–	6.67*	7.9	5.7	16.0	11.44**	6.9	3.0	10.1	9.40**	4.1	7.1	8.3	3.12
Substance dependence in the household	12.8	2.8	16.7	12.01**	15.1	8.7	24.5	19.77***	17.0	7.6	17.3	16.94***	12.1	12.8	4.2	1.47
Mental illness in the household	6.6	0.7	5.9	7.33*	9.8	2.1	2.2	31.26***	10.7	3.7	10.1	14.17***	12.2	5.0	8.7	6.35*
Prison stay	2.6	2.1	5.6	0.78	2.5	1.9	4.3	1.86	3.8	2.0	6.5	5.51	2.2	3.5	8.3	4.11

***p < 0.001, **p < 0.01, *p < 0.05.

Significant differences in the frequencies of ACE within West and East Germany and abroad were found primarily in the cohorts 1946–1967 and 1968–1989. Emotional abuse, physical abuse, emotional and physical neglect, and a household member's substance dependence as well as mental illness show highly significant differences (*p* =< 0.001). Respondents who spent most of their childhood and adolescence in East Germany report less emotional abuse, physical abuse, neglect, and substance dependence. The mental illness of a household member is most frequently written by persons who grew up predominantly in West Germany. Participants born up to 1945 show highly significant differences only in the case of separation of a parent, especially among East Germans; the number is 29.4%, significantly higher than the comparison groups (*p* =< 0.001). Physical neglect is highly significant in the youngest age cohort (*p* =< 0.002). Here, West Germans report less physical neglect than persons who grew up predominantly abroad (*p* =< 0.002). Physical and sexual abuse, emotional neglect, and mental illness of a household member, also show significance (*p* =< 0.05).

### Predictors of experiencing adverse childhood experiences

The logistic regression results are shown in Table 5. Sociopolitical context emerges as a predictor across almost all ACEs. An increased likelihood of experiencing adverse childhood experiences among those raised abroad is especially evident for the ACEs of emotional and physical abuse [odds ratio (OR) = 1.693, *p* =< 0.001; OR = 2.208, *p* =< 0.001), and physical neglect (OR = 2.920, *p* =< 0.001). Coming from East Germany decreases the likelihood of having experienced adverse childhood experiences.

**Table 5:** Binary logistic regression to examine the association between adverse childhood experiences (dependent variable^1^) and gender, origin, and age (independent variables) for the entire sample (*N* = 5,018) as odds ratios and 95% confidence intervals.

	**Gender Ref = Male**	**Raised** **Ref** = **West Germany**		**Age cohorts** **Ref** = **Until 1945**		
	**Female**	**East Germany**	**Abroad**	**1946–1967**	**1968–1989**	**From 1990**
Emotional abuse	0.94 [0.80–1.09]	0.43*** [0.34–0.55]	1.69*** [1.26–2.22	1.05 [0.89–1.41]	0.88 [0.66–1.19]	0.69* [0.49–0.96]
Physical Abuse	0.75** [0.63–0.90}	0.49*** [0.37–0.64]	2.20*** [0.37–2.96]	0.86 [0.64–1.18]	0.68 [0.50–0.93]	0.57 [0.40–0.82]
Sexual Abuse	4.07*** [2.86–5.94]	0.49** [0.29–0.78]	1.17 [0.63–1.99]	2.26* [1.18–4.89]	2.28* [1.19–4.94]	1.96 [0.96–4.43]
Emotional neglect	1.07 [0.91–1.26]	0.46*** [0.35–0.59]	1.00 [0.70–1.40]	1.00 [0.74–1.36]	1.04 [0.77–1.42]	0.79 [0.56–1.12]
Physical neglect	0.95 [0.72–1.24]	0.40*** [0.24–0.61]	2.92*** [1.95–4.26]	0.48*** [0.33–0.72]	0.40*** [0.27–0.61]	0.29*** [0.17–0.48]
Separation from a parent	0.98 [0.84–1.13]	1.02 [0.85–1.22]	0.95 [0.69–1.30]	0.87 [0.67–1.14]	1.19 [0.92–1.56]	1.16 [0.87–1.56]
Violence against the mother	0.97 [0.77–1.21]	0.64** [0.46–0.87]	1.67** [1.10–2.45]	1.02 [0.73–1.53]	0.81 [0.55–1.22]	0.59 [0.63–0.95]
Substance dependence in the household	1.12** [1.06–1.47]	0.51*** [0.40–0.65]	1.24 [0.89–1.69]	1.46 [1.06–2.06]	1.56** [1.12–2.20]	1.17 [0.81–1.71]
Mental illness in the household	1.13 [0.92–1.38]	0.26*** [0.17–0.38]	0.65 [0.38–1.02]	1.50* [1.02–2.02]	1.90** [1.23–3.10]	2.19** [1.37–3.63]
Prison stay	0.92 [0.66–1.28]	0.74 [0.45–1.17]	1.95* [1.09–3.27]	0.92 [0.50–1.85]	1.33 [0.74–2.63]	0.97 [0.48–2.05]

^1^Reference category = “No.”

Odds ratio (OR) and 95% confidence interval (95% CI); ***p < 0.001, **p < 0.01, *p < 0.05.

The bold values show the significant values.

A strong negative influence on physical neglect is evident between all age cohorts (1946–1967: OR = 1.590, *p* = > 0.001; 1968–1989: OR = 1.1909, *p* =< 0.001; from 1990: OR = 2.190, *p* =< 0.001).

Women were more likely to be victims of sexual abuse (OR = 4.071, *p* =< 0.001) and less likely than men to be at risk of physical abuse (OR = 0.755, *p* =< 0.002).

## Discussion

In the present study, we examined the prevalence of individual ACEs and the likelihood of experiencing abuse, neglect, and household dysfunction in dependence on age and gender and where childhood and youth were spent predominantly. In addition to gender and age, sociopolitical context predicted nearly all the ACEs examined. Where someone grew up proved to be the strongest predictor of experiencing adverse childhood experiences. As the first study on a German representative sample, our results show that the risk of experiencing ACEs is lower for participants from East Germany than those from West Germany or abroad.

There are many possible reasons for the above-described findings. Household dysfunction or economic inequality and precarity are considered social risk factors for abuse in childhood and adolescence, of which the former GDR was less affected (18, 39, 40). Childcare may also play a possible role in the frequency of abuse experiences. For example, there were state-directed policies and structures such as out-of-home care facilities and other collective institutions in the former GDR by 1965. Thus, children's growing up took place more in social networks, which proved to be a protective factor against ACEs (28). In their studies Gordon et al. (41) refer to the association between high-quality childcare and low symptoms of maternal depression.

In contrast, childcare in West Germany took place more within the family system and was thus more dependent on socioeconomic opportunities and family dynamics. This could be why children in West Germany were more affected by abusive and harmful family structures (42). However, there is also a downside to it; The state's educational function in the former GDR led to institutional violence, especially in youth welfare facilities. On the one hand, this was due to the excessive demands on the educators to ensure compliance with the norms in the parental home, which was often solved by force.

Our results regarding sexual abuse in East and West Germany are in line with the findings of Schötensack et al. (33) and Ulke et al. (32). Sexual abuse was reported more frequently in the age cohorts 1946–1989. However, no differences can be identified before and after establishing the GDR.

A lack of awareness of sexual violations of boundaries is caused by socially propagated permissive sexual education. Thus, sex education in the GDR was seen as part of personality education, which was the responsibility of all state educational institutions (43). From an epidemiological perspective, however, these cases of affected children were minor. They are also unlikely to have occurred more frequently than other forms of institutional violence in West Germany, such as in Catholic boarding schools or Welfare institutions for children's recovery or prevention. Dreßing et al. (44) showed that sexual abuse happened quite frequently within the catholic clergies. Since the catholic church did not have much power and societal interweaving in the former GDR, it might be that this may be an explanation for the higher prevalence rates in West Germany.

Another German cohort study shows that parental separation may be associated with psychological impairment in adulthood (45). However, the results of our study show no significant difference for separation from one parent. This suggests that children in the former GDR have not felt the separation of a parent more often than children in the FRG.

Findings by Austin et al. (16) suggest that sociopolitical policies can reduce child maltreatment rates. For example, corporal punishment was banned in the former GDR as early as 1949. A study on remembered parental parenting behavior underpins this assumption: People from East Germany reported significantly less rejection and punishment by mother and father than the West German comparison group (46). In West Germany, corporal punishment was still tolerated until 1973, and in Bavaria until 1983. This could express a greater acceptance of physical discipline strategies in West Germany, associated with greater use of physical violence against children and higher prevalence rates (47). This view can be confirmed by the dramatic decrease in corporal punishment in Sweden after the ban of this educational measure in 1979 (48). Germany also showed a significant decrease in corporal punishment against children and adolescents after children's right to a non-violent upbringing was enshrined in law in the Civil Code in 2000 (15). Another reason for more violent and abusive parenting behavior could be the greater acceptance of parenting advice widely used in the “Third Reich,” such as the parenting books by Johanna Haarer. Such books were banned in the former GDR. For example, the author (49) recommends leaving the child alone in a room day and night, not responding to the child's crying, limiting food intake, and starving the child if necessary.

Highly pronounced maternal depressive symptomatology is a risk factor for child physical abuse and neglect (50–52). Adverse effects and emotions have a significant influence on the child's development. Thus, self-experienced negative childhood experiences of the mother can be directly related to internalized and externalized difficulties in the children and thus complicate the positive mother-child relationship (53). Another risk factor for childhood abuse seems to be the wellbeing of the woman and mother, which is related, among other things, to the opportunity for education and gender equality (23, 54). Thus, there is a broad consensus that improving the position of women and mothers positively impacts the wellbeing of their children (17, 20, 21). The importance and impact of maternal wellbeing on child health are illustrated by an experimental study by Maccari et al. (55); for example, stress during pregnancy decreases motherly guidance during early childhood, which can have lifelong effects on emotional behavior and increased susceptibility to age-related disorders.

Almost all ACEs decreased with the increasing birth year of the respondents. For example, physical abuse was reported in 13.3% of those born up to 1945 and decreased to 8.9% in the 1990 and older age cohort. The increase could be due to the growing awareness of these societal issues (56). However, the results are striking on whether a household member was depressed, mentally ill, or had ever attempted suicide. This shows a significant increase from 4.7 to 10.9% between all age cohorts [χ ^2^(3) = 18,05; *p* =< 0.001]. There are numerous reasons for this; social inequality, higher workloads, and increased sensitivity are just some of them (57, 58). Interestingly the prevalence differences of ACEs between East and West Germany almost disappeared in the generations born after the unification. This finding supports our hypothesis of the importance of socio-political factors in the occurrence of ACEs, as with an alignment of socio-political environments after the unification, the prevalence rates of ACEs also do align.

## Conclusion

Adverse childhood experiences are a fundamental public health problem. In order to ensure a safe and stable environment for children, it is crucial to consider societal circumstances and influences in addition to family risk factors. The role of motherhood in society and politics should also be emphasized here (23, 51). Thus, the mother's wellbeing is positively related to adverse childhood experiences. This aspect can be of great importance for preventing child abuse and neglect as well as for the treatment, e.g., psychotherapeutic support of the mother. A large body of research confirms the effectiveness of various interventions regarding adverse childhood experiences at individual and family levels (59, 60). However, it can be assumed that prevention interventions focusing on social-political risk factors would significantly affect the prevalence of adverse childhood experiences.

## Limitations and strengths

Our study has several strengths, including the use of nationally representative data. Also noteworthy is the precise description of the different items for adverse childhood experience. However, it cannot be excluded that cultural or contextual differences played a role in the response behavior and may have impacted prevalence rates.

However, some limitations of the present research must also be considered. For example, the available data do not provide precisely where a person spent childhood and adolescence exactly. This is primarily a limitation for the participants who were raised abroad since we cannot further differentiate the participant's countries and regions. Also, internal migration was not considered in the present work. Domestic isolation might have had an impact on the psychological condition and thus might have had an effect on the response behavior of the respondents. Memory and social desirability biases may be limited data on severe abuse and neglect (61). Furthermore, the data collection period should be mentioned as a possible limitation of the study, which was collected in the first Covid wave.

## Data availability statement

The raw data supporting the conclusions of this article will be made available by the authors, without undue reservation.

## Ethics statement

The studies involving human participants were reviewed and approved by Ethics Committee of the Medical Faculty of Leipzig University. Written informed consent to participate in this study was provided by the participants' legal guardian/next of kin.

## Author contributions

A-CS and EB designed and directed the project. A-CS processed the data, performed the analysis, drafted the manuscript, and designed the figures. CK and EB contributed to the interpretation of the results. EB, CK, MB, JF, and VC contributed to the writing of the manuscript and supervised the work. All authors contributed to the article and approved the submitted version.

## Conflict of interest

The authors declare that the research was conducted in the absence of any commercial or financial relationships that could be construed as a potential conflict of interest.

## Publisher's note

All claims expressed in this article are solely those of the authors and do not necessarily represent those of their affiliated organizations, or those of the publisher, the editors and the reviewers. Any product that may be evaluated in this article, or claim that may be made by its manufacturer, is not guaranteed or endorsed by the publisher.
